# Health outcomes and healthcare delivery systems for pregnant and postpartum individuals in immigration detention settings: A scoping review protocol

**DOI:** 10.1371/journal.pone.0352692

**Published:** 2026-06-26

**Authors:** Elizabeth Kravitz, Daniel Delmonaco, Chelsea A. DeBolt

**Affiliations:** 1 Department of Obstetrics and Gynecology, Division of Maternal Fetal Medicine, Icahn School of Medicine at Mount Sinai, New York, New York, United States of America; 2 Icahn School of Medicine at Mount Sinai, New York, New York, United States of America; Sun Yat-Sen University, CHINA

## Abstract

Pregnant and postpartum individuals in immigration detention may experience barriers to accessing timely and appropriate perinatal care. The existing literature remains fragmented across global settings. This scoping review protocol aims to map the extent, range, and nature of evidence on health outcomes and healthcare delivery systems for pregnant and postpartum individuals in immigration detention. Following the Arksey and O’Malley framework and PRISMA-ScR guidelines, peer-reviewed studies and relevant grey literature will be included without language restrictions, after January 2000. Two reviewers will independently screen sources, and a third will perform full text review and extract data. Findings will be synthesized descriptively and thematically.

## Introduction

As of June 2025, an estimated 117.3 million people are forcibly displaced [1]. Immigration detention is increasingly used as a migration management strategy, including for individuals who are pregnant [2,3]. Pregnant individuals in immigration detention face unique and significant barriers to accessing quality perinatal care, with evidence suggesting increased risk for adverse maternal and neonatal outcomes, discrimination, and fragmented care delivery [4–7]. Standards governing healthcare in immigration detention vary widely across countries, and in some settings, including the United States, there are limited standardized guidelines and enforcement mechanisms [8,9]. Furthermore, the definition of “pregnancy care” varies widely in the literature and globally [10]. The literature is heterogeneous, with inconsistent definitions and limited synthesis of global data, particularly regarding the intersection of immigration status, incarceration, and pregnancy [4–7,10,11]. While prior work has examined pregnancy outcomes and healthcare delivery for pregnant individuals in criminal justice custody and for undocumented pregnant immigrants living in the community, to our knowledge, no comprehensive global synthesis exists at the intersection of immigration detention and prenatal healthcare delivery [12].

Given the variation in immigration detention practices globally and the lack of standardized healthcare delivery and outcomes reporting, a scoping review is warranted to map the available evidence and identify key knowledge gaps. Immigration detention facilities lack transparency, and this research protocol addresses a critical public health and human rights issue affecting a vulnerable, growing population [13].

This scoping review will aim to: (1) map the extent, range, and nature of evidence on health outcomes and healthcare delivery systems for pregnant and postpartum individuals in immigration detention settings; (2) describe barriers and facilitators to care, models of service delivery, and structural or policy-related factors; and (3) identify gaps to inform future research, policy, and practice.

## Methods

This protocol follows the Arksey and O’Malley scoping review framework with enhancements from the Joanna Briggs Institute and PRISMA-ScR guidelines [14,15]. The review is informed by reproductive justice and candidacy frameworks, applied as analytic tools to guide data extraction and thematic synthesis regarding healthcare access and delivery in immigration detention settings. The protocol has been registered with Open Science Framework (OSF) and can be accessed online at https://osf.io/m96jd/overview (Registration DOI https://doi.org/10.17605/OSF.IO/M96JD) [16].

We will include peer-reviewed studies and grey literature involving pregnant and/or postpartum individuals detained in immigration detention or similar custodial settings globally. Quantitative, qualitative, mixed-methods studies, reviews, and grey literature will be eligible. Grey literature will include government reports, policy briefs, theses and dissertations, and publications from non-governmental and international organizations (e.g., advocacy reports, technical documents, and oversight reports). Theses and dissertations will be included when they meet eligibility criteria and contain sufficient methodological detail and relevant data on pregnancy-related health outcomes or healthcare delivery in immigration detention settings. No language restrictions will be applied. Searches will be limited to sources published from January 2000 onward, reflecting the expansion of contemporary immigration detention systems internationally and the evolution of modern standards of perinatal care and health reporting [17–19]. Inclusion criteria will include sources describing pregnant and/or postpartum individuals in immigration detention or comparable custodial immigration settings. Sources will be included if they report on health outcomes, healthcare delivery systems, access to care, and/or barriers and facilitators to pregnancy-related care. Exclusion criteria include sources focused exclusively on non-detained immigrant populations, sources limited to incarceration in criminal justice settings without an immigration detention component, and articles that do not address pregnancy-related health outcomes or healthcare delivery. Further, opinion pieces, commentaries, or media reports without original data or substantive analysis will also be excluded.

Peer-reviewed databases will include PubMed, Scopus, Web of Science, CINAHL, EMBASE, Global Health (Ovid), and Global Index Medicus (Table 1). Appendix 1 contains the full search strategy for each database that will be included in this review. Search results will be imported into Covidence software, and duplicates will be removed prior to screening [20]. Screening and full text review of the peer-reviewed literature is estimated to be completed by two independent reviewers with a third reviewer available to resolve discrepancies during screening and full-text review. Data charting will be conducted using a standardized extraction form developed for this review. The form will be pilot-tested on a subset of included studies and iteratively refined to ensure clarity and consistency. After determination of the final texts to be included in the study, data will be extracted by one reviewer and verified by a second reviewer for accuracy and completeness. This data will be review by the full authorship team. All data will be screened, reviewed, and extracted on Covidence software [20].

**Table 1:** Summary of search strategies by database. Search terms (simplified for presentation; full Boolean search strategies, including synonyms and controlled vocabulary terms, are provided in S1 Appendix).

Database	Search terms (simplified, for full report see Appendix 1)
PubMed	pregnan* OR postpartum AND immigrant* AND detention
Scopus	TITLE-ABS-KEY(pregnan* AND immigrant* AND detention)
Web of Science	TS=(pregnan* AND immigrant* AND detention)
CINAHL	MH Pregnancy AND MH Immigrants AND MH Detention
EMBASE	exp pregnancy/ AND exp immigrant/ AND exp detention/

Grey literature searches will be limited to sources published from January 2000 onward and will include materials in all languages. For sources yielding a large number of results (e.g., >100 records), a staged screening approach will be used. An initial subset of the first 50 records will be screened to assess yield. If ≥20% of these records meet inclusion criteria and are non-duplicate, the full set of results will be screened; otherwise, screening will be restricted to the initial subset. Grey literature will be screened by one reviewer and verified by a second reviewer for eligibility and inclusion, with discrepancies resolved through discussion and, if needed, consultation with a third reviewer. The grey literature will be reviewed within a three-month period to minimize variability in the literature extracted. Grey literature will be identified through WorldCat, GovInfo, Refworld, and organizational websites (Table 2) and stored on a password protected excel spreadsheet.

**Table 2:** Grey literature sources and search approach.

Source	Type of material	Search approach and relevance
WorldCat	Government reports, NGO publications, policy documents	Keyword searches combining pregnancy, immigration, and detention to identify global grey literature, including government oversight and NGO reports
GovInfo	U.S. government reports and oversight documents	Searches of GAO, DHS, and congressional materials related to immigration detention, healthcare provision, and pregnancy
Refworld	Human rights reports and legal documentation	Identification of international reports on immigration detention, migrant health, and reproductive health outcomes
Transactional Records Access Clearinghouse (TRAC)	Administrative and oversight reports	Review of reports and datasets related to immigration detention practices and oversight
Physicians for Human Rights	Human rights investigations and medical-legal reports	Screening of reports documenting health conditions, access to care, and treatment in detention settings
United Nations High Commissioner for Refugees (UNHCR)	Global policy reports and epidemiologic summaries	Identification of reports addressing displacement, detention practices, and health outcomes
World Health Organization (WHO)	Global health guidance and technical reports	Review of guidance and reports related to migrant health, detention, and maternal care
Amnesty International/ Human Rights Watch	Investigative reports and advocacy documentation	Screening of reports addressing conditions of detention and access to healthcare for detained populations

The study team will extract data including contextual characteristics, as well as pregnancy-related health outcomes and healthcare delivery characteristics. Data extraction will be informed by reproductive justice and candidacy frameworks, which will guide the extraction of variables related to healthcare access, care delivery, barriers and facilitators, structural and policy factors, and patient autonomy within detention settings [21,22]. Reproductive justice will inform extraction of data related to bodily autonomy, the ability to continue pregnancy and access pregnancy-related care, and the role of state or institutional control, while candidacy will be used to identify how care needs are recognized, navigated, adjudicated, offered, resisted, or constrained by operating conditions. During synthesis, a hybrid thematic analysis approach will be used, in which these framework-informed domains serve as deductive coding categories alongside inductive coding to capture emergent themes. Findings will then be organized thematically to examine how structural conditions of immigration detention shape access to perinatal care and reported maternal and neonatal outcomes (Table 3).

**Table 3:** Data extraction domains and variables.

Domain	Variables Extracted
Study characteristics	Study design, publication type, year, funding source
Population characteristics	Pregnancy/postpartum status, demographics (if reported)
Detention context	Country, type of detention setting, definitions used
Structural/policy context	Relevant policies, oversight structures
Healthcare delivery models	Type and organization of care, services available
Access to care	Timing, availability, referral pathways
Barriers and facilitators	Structural, institutional, and individual-level factors
Perinatal health outcomes	Maternal and neonatal outcomes
Theoretical framework domains	Reproductive justice (e.g., autonomy, right to parent); candidacy (e.g., identification, navigation, adjudication)
Key findings	Summary of study findings

Contextual characteristics will include population characteristics, definition of pregnancy and detention used, country/countries discussed in text, type of detention center, legal/policy environment, and models of healthcare delivery. Additional variables will include study design and publication type, publication year, and funding source, if any. Pregnancy health outcomes and healthcare delivery characteristics will include models of care, barriers and facilitators to care, access to services, maternal and neonatal health outcomes, and key findings. Results will be synthesized descriptively and thematically. Results are expected by August 2026.

## Discussion

This scoping review will provide the first comprehensive mapping of global evidence on perinatal health outcomes, access to care, and healthcare delivery models for pregnant and postpartum individuals in immigration detention settings. By synthesizing findings from both peer-reviewed studies and relevant grey literature, the review will clarify the current state of the evidence, including how outcomes are defined and measured, how care is organized and delivered in different detention contexts, and what barriers and facilitators to care have been identified. The application of reproductive justice and candidacy frameworks will support an analytic understanding of how structural, policy, and contextual factors shape access to and engagement with healthcare services in detention, offering insight into mechanisms that may contribute to variation in care experiences and health outcomes. Identifying areas of concentration and gaps in the literature will help guide future research priorities, support improvements in reporting and data standards, and inform policymakers, practitioners, and researchers interested in improving perinatal care in settings of immigration custody. As immigration detention practices and health systems continue to evolve globally, this review will contribute a foundational evidence base upon which future work will be built. These findings will also highlight global inequities in access to perinatal care among detained populations, particularly across different legal, economic, and healthcare system contexts. Findings from this review will have direct implications for policy by identifying gaps in standards, oversight, and delivery of perinatal care in immigration detention settings, which may inform the development of more equitable and evidence-based guidelines.

This protocol has several limitations. First, although a comprehensive search of bibliographic databases and grey literature sources is planned, news media sources will not be systematically searched and may contain additional contextual information on pregnancy and healthcare in immigration detention settings. Second, definitions of immigration detention, custodial settings, and pregnancy-related outcomes are likely to vary across sources, limiting comparability across studies. Third, much of the available evidence is expected to derive from grey literature, which may differ in methodological rigor, reporting standards, and completeness of outcome data. Fourth, evidence may be geographically concentrated in certain high-income countries, particularly the United States, with limited representation from other global regions. Consistent with scoping review methodology, we will not conduct formal critical appraisal of included sources, and findings should therefore be interpreted as descriptive mappings of the existing literature rather than assessments of effect size or study quality. Finally, given the dynamic nature of immigration and detention policies, findings may not reflect current practices in all settings.

## Supporting information

S1 Appendix Full Search Strategies by Database. (DOCX)

S1 PRISMA-SR Checklist Preferred Reporting Items for Systematic reviews and Meta-Analyses extension for Scoping Reviews (PRISMA-ScR) Checklist. (DOCX)
