# Supplementary material for: Health outcomes and healthcare delivery systems for pregnant and postpartum individuals in immigration detention settings: A scoping review protocol
Source: PLoS One. 2026 Jun 26;21(6):e0352692. doi: 10.1371/journal.pone.0352692 (PMC13308821; doi:10.1371/journal.pone.0352692)
Supplement: S1 Appendix — (DOCX) [file pone.0352692.s001.docx]

# S1 Appendix 1. Full Search Strategies by Database

## PubMed

("Pregnant People"[Mesh] OR "Pregnancy"[Mesh] OR "Parturition"[Mesh] OR "Prenatal Diagnosis"[Mesh]
OR prenatal OR pregnan* OR postpartum OR birth*)
AND
("Undocumented Immigrants"[Mesh] OR "Emigrants and Immigrants"[Mesh] OR "Refugees"[Mesh]
OR undocumented OR immigrant OR immigrat* OR asylum OR refugee OR migrant)
AND
("Jails"[Mesh] OR "Quarantine"[Mesh] OR "Correctional Facilities"[Mesh]
OR "immigrant detention" OR "detention center" OR "immigration and customs"
OR "homeland security" OR "holding facilit*" OR detain* OR prison OR jail
OR imprison* OR incarcerat* OR "carceral setting" OR penal OR internment)

## Scopus / Web of Science / Global Index Medicus

(Parturition OR Prenatal OR pregnan* OR postpartum OR birth*)
AND
(Undocumented OR immigrant OR immigrat* OR asylum OR refugee OR migrant)
AND
("immigrant detention" OR "detention center" OR "immigration and customs"
OR "homeland security" OR "holding facilit*" OR detain* OR prison OR jail
OR imprison* OR incarcerat* OR "carceral setting" OR penal OR internment
OR "correctional facilit*")

## CINAHL

(MH "Labor+" OR MH "Expectant Mothers" OR MH "Pregnancy+" OR MH "Prenatal Care")
AND
(MH "Undocumented Immigrants" OR MH "Immigrants+" OR MH "Refugees+")
AND
(MH "Correctional Facilities" OR MH "Incarceration" OR "immigrant detention"
OR detain* OR incarcerat*)

## EMBASE

(exp birth/ OR exp prenatal care/ OR exp prenatal diagnosis/ OR exp pregnant person/)
AND
(exp undocumented immigrant/ OR exp migrant/ OR exp refugee/ OR asylum seeker/)
AND
(exp immigration detention/ OR exp detention center/ OR exp correctional facility/
OR exp prisoner/)
